# Non-tubal ectopic pregnancy: types of treatment and occurrence of severe complications in a university hospital

**DOI:** 10.61622/rbgo/2025rbgo70

**Published:** 2025-10-21

**Authors:** Arthur Chaves de Almeida, Ricardo Ruiz Garcia de Almeida, Barbara Bizzo Castelo, Luiz Francisco Cintra Baccaro

**Affiliations:** 1 Universidade Estadual de Campinas Campinas SP Brazil Universidade Estadual de Campinas, Campinas, SP, Brazil.

**Keywords:** Pregnancy complications, Pregnancy, ectopic, Pregnancy trimester, first

## Abstract

**Objective:**

To evaluate the types of treatments used for non-tubal ectopic pregnancy (NTEP), the success rates of medical treatment, and the incidence of severe complications.

**Methods:**

Retrospective study of all NTEP admitted in the University of Campinas (UNICAMP) Women's hospital, Brazil, from 01/01/2000 to 01/31/2023. Outcome variables were medical treatment success and the presence of severe complications. Independent variables were clinical and sociodemographic data. Statistical analysis was carried out by the Cochran–Armitage and chi-square test.

**Results:**

In total 60 cases of NTEP were included (3 abdominal, 15 cervical, 12 cesarean scar, 24 interstitial fallopian tube, 3 heterotopic e 3 ovarian). In cases of abdominal, cesarean scar, heterotopic and ovarian NTEP, the main type of treatment was surgery. Medical treatment was used mainly in cervical and interstitial NTEP (p<0.01). Medical treatment (included methotrexate single dose, multiple doses or direct injection) success rate was 73.7%, with no correlation with NTEP location (p=1.000). No change in trend was identified in the success rate of medical treatment for NTEP in the period evaluated (Cochran-Armitage test: z=0.46; p= 0.644). Severe complications occurred in 12(20) of NTEP, with no association with NTEP location (p=0.27), but with hemodynamic instability (p=0.01).

**Conclusion:**

The medical treatment success rate found in our study for NTEP was similar to that reported in the medical literature for tubal EP. Two out of ten women with NTEP had severe complications. The occurrence of severe complications was not associated to the site of NTEP but to the presence of hemodynamic instability at admission.

## Introduction

An ectopic pregnancy (EP) occurs when the blastocyst implants in a location other than the uterine cavity. EP is the leading cause of maternal death in the first trimester of pregnancy.^(1)^ It accounts for approximately 2% of all pregnancies.^(1)^ In most cases, the fallopian tube is the most common implantation site, representing 95% of cases.^(1)^ However, other possible sites include the ovaries, uterine scar, intestinal loops, cervix, and interstitial portion of the fallopian tube. Although less frequent, non-tubal ectopic pregnancy (NTEP) are associated with a higher maternal risk.^(2)^

Interstitial EP occurs when implantation takes place in the segment of the fallopian tube embedded within the myometrium, a thin region prone to early rupture and significant hemorrhage.^(3)^ When implantation occurs in the fibrotic tissue of a previous hysterotomy, it results in a cesarean scar EP. A rarer site of implantation is the cervix, accounting for less than 1% of ectopic pregnancies. Ovarian pregnancy is considered a sporadic event with distinct risk factors compared to other types of EP. Abdominal EP can be classified as primary—when implantation occurs directly on the peritoneal surface—or secondary—when the fertilized egg initially implants in the fallopian tube and subsequently migrates to the peritoneal cavity.^(3)^

EP treatment can be either medical or surgical, depending on the patient's clinical condition and the characteristics of the EP. Medical management involves the administration of methotrexate (MTX), with follow-up monitoring of human chorionic gonadotropin (HCG) levels and the patient's overall condition. Treatment protocols vary, classified as either long or short, depending on the number of MTX doses administered.^(4)^ MTX avoids the risks and complications of surgery; however, it is only suitable for clinically stable patients with an early diagnosis of EP. Conversely, surgical treatment involves either the removal of the gestational sac or the excision of the surrounding tissue where implantation occurred. This can be performed via laparoscopy or laparotomy, depending on the patient's condition, the hospital's resource availability, and the expertise of the medical team.

Limited data are available on NTEP and the effectiveness of different treatment modalities. The University of Campinas (UNICAMP) Women's Hospital is a tertiary care center located in Campinas - SP, southeastern Brazil. It receives pregnancy-related complication cases from multiple cities in the region. This study aimed to evaluate the frequency of NTEP over the past 23 years, the treatment approaches used, the success rate of medical management, the incidence of severe complications, and the associated risk factors.

## Methods

We conducted a retrospective observational study using data from all women admitted to the UNICAMP Women's Hospital between January 1, 2000, and January 31, 2023, who were diagnosed with NTEP. Cases were identified using the following International Classification of Diseases, 10th Revision (ICD-10) codes: O00 (ectopic pregnancy); O00.0 (abdominal pregnancy); O00.1 (tubal pregnancy); O00.2 (ovarian pregnancy); O00.8 (other ectopic pregnancies); and O00.9 (unspecified ectopic pregnancy). Data collection was conducted by the researchers through a thorough review of medical records in the hospital's Medical Archive and Statistics Service. Cases that were not confirmed as ectopic pregnancies or were classified as tubal EP were excluded from the study.

- Successful medical treatment for NTEP: defined as cases in which women treated with methotrexate did not require subsequent surgical intervention. Patients were monitored through serial hCG measurements until negative. Cases lost to follow-up before achieving biochemical resolution were classified as having an unknown outcome, as surgical intervention may have been performed at another facility.
- Severe complications: defined as the occurrence of any of the following during hospitalization: blood transfusion, Intensive Care Unit (ICU) admission, surgical reintervention, hysterectomy, or death due to ectopic pregnancy.

We considered the independent variables to be as follows: the year of occurrence of ectopic pregnancy; EP location; the mean diameter of gestational sac of the ectopic pregnancy on ultrasound; quantitative serum hCG at diagnosis (measured in mUI/mL); embryonic heartbeat on ultrasound; gestational age at diagnosis (calculated by the date of the last menses and by ultrasound analysis when available); EP integrity while the diagnoses and during evolution; woman's age; weight; height; body surface; body mass index (BMI – body mass index, calculated as weight in kilograms divided by the square of height in meters); skin color; educational level; marital status; parity; previous history of ectopic pregnancy; history of tubal ligation; history of pelvic inflammatory disease; history of surgical procedures such as laparotomy or laparoscopy; history of intrauterine device as a contraceptive method as well as its use during the diagnosis of ectopic pregnancy; symptoms reported when seeking emergency care (abdominal pain, vaginal bleeding, absence of symptoms, or other symptoms reported); smoking and current pregnancy resulting from in vitro fertilization; and methotrexate dose administered.

Statistical analysis began with a descriptive assessment of the data. Categorical variables were summarized in tables with absolute (n) and relative (%) frequencies, while continuous variables were expressed as mean, standard deviation, median, minimum, and maximum values.

To analyze trends in annual clinical treatment success rates, the Cochran-Armitage test for trend was applied. Comparisons between categorical variables (successful medical treatment and severe complications) were performed using the χ² test or Fisher's exact test, as appropriate. For continuous variables, the Mann–Whitney test was applied due to the non-normal distribution of data. Statistical significance was set at 5%. All analyses were conducted using the Statistical Analysis System for Windows, version 9.2 (SAS Institute Inc., 2002–2008).

The research project was submitted to and approved by the UNICAMP Research Ethics Committee 6.102.993 (*Certificado de Apresentação de Apreciação Ética*: 53019116.6.0000.5404).

## Results

We identified a total of 966 cases of EP during the study period. Of these, 60 cases were classified as NTEP and were included in the analysis. The median age of the women was 29.97 ± 6.59 years, and the median body mass index (BMI) was 26.69 ± 6.73. The clinical and sociodemographic profiles of these women are detailed in table 1.

**Table 1:** Sociodemographic and obstetrics characteristics of NTEP cases

Variables		n(%)
Grouped years		
	2000 - 2007	8(13.33)
	2008 - 2015	21(35)
	2016 - 2022	31(51.67)
Age		
	<20	5(8.33)
	20 - 29	20(33.33)
	30 - 39	32(53.33)
	40 - 49	3(5)
Level of education*		
	None	7(23.33)
	Elementary school - incomplete	2(6.67)
	Elementary school - complete	8(26.67)
	High school - incomplete	2(6.67)
	High school complete	7(23.33)
	Undergraduate degree	4(13.33)
Marital status*		
	With partner	31(67.39)
	No partner	15(32.61)
Skin colour*		
	White	42(75)
	Black	4(7.14)
	Brown	10(17.86)
Number of previous pregnancies*		
	0	14(23.33)
	1	15(25)
	≥2	17(51.67)
Number of previous cesarean deliveries		
	0	34(56.67)
	1	19(31.67)
	≥2	7(11.66)
Number of previous abortions		
	0	36(60)
	1	17(28.33)
	≥2	7(11.67)
Previous ectopic pregnancy		
	No	54(90)
	Yes	6(10)
Smoking*		
	No	42(79.25)
	Yes	11(20.75)

* Missing cases

Most women had some symptoms upon admission, with 10(16.67) having only abdominal pain, 14(23.33) having only vaginal bleeding and 21(35) having abdominal pain associated with bleeding vaginal. The mean gestational age by amenorrhea was 8.07 ± 2.56 weeks, gestational age by ultrasound was 8.50 ± 3.49 weeks, mean gestational sac diameter was 36.72 ± 20.49 mm, serum HCG was 15,573 ± 23,705 mIU/ml. Regarding the location of the ectopic pregnancy: abdominal 3(5), cervical 15(25), cesarean section scar 12(20), interstitial 24(40), heterotopic 3(5), ovarian 3(5). Other characteristics presented at admission can be found in table 2.

**Table 2:** Clinical characteristics of NTEP cases

Variables		n(%)
Ectopic pregnancy location		
	Abdominal	3(5)
	Uterus cervix	15(25)
	Cesarean scar	12(20)
	Interstitial fallopian tube	24(40)
	Heterotopic	3(5)
	Ovarian	3(5)
Ectopic pregnancy integrity		
	No rupture	42(71.19)
	Rupture	17(28.81)
	Ignored or unavailable	1
Embryonic heartbeat on ultrasound		
	No	44(75.86)
	Yes	14(24.14)
	Ignored or unavailable	2
Symptons at admission		
	Asymptomatic	15(25)
	Only abdominal pain	10(16.67)
	Only vaginal bleeding	14(23.33)
	Abdominal pain and vaginal bleeding	21(35)

The initially proposed type of treatment varied significantly depending on the location of the EP (p<0.01). In cases of abdominal, cesarean scar, heterotopic, and ovarian EPs, surgery was the primary treatment approach, with conservative procedures being the most common. However, hysterectomy was required in two cases of cesarean scar pregnancy and one case of abdominal pregnancy. Medical treatment was primarily used for cervical and interstitial EPs, with single-dose intramuscular MTX being the most frequently administered option. However, two cases of cervical ectopic pregnancy were treated with direct injection of MTX, while multiple-dose MTX was used in two interstitial cases and one cervical case. The distribution of cases and treatment groups is presented in table 3.

**Table 3:** Type of treatment proposed and location of EP

EP location		Abdominal n(%)	Uterus cervix n(%)	Cesarean scar n(%)	Interstitial n(%)	Heterotopic n(%)	Ovarian n(%)
Surgical		3(100)	3(20)	10(83.3)	13(54.2)	2(66.6)	3(100)
	Hysterectomy	1(33.3)	0	2(16.6)	0	0	0
	Conservative	2(66.6)	3(20)	8(66.6)	13(54.2)	2(66.6)	3(100)
Clinical		0	12(80)	2(16.67)	10(41.6)	1(33.3)	0
	Single dose	0	10(66)	1(8.3)	8(33.3)	1(33.3)	0
	Multiple doses	0	0	1(8.3)	2(8.3)	0	0
	Direct injection	0	2(14)	0	0	0	0
Expectant		0	0	0	1(4.2)	0	0

Fisher's exact test: p = 0.006

**Figure 1:**
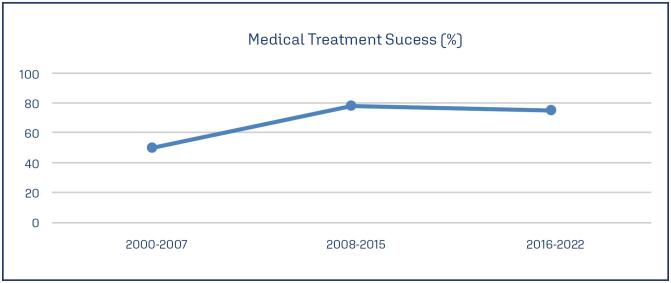
Medical treatment success along the years

Regarding the success of medical treatment, six out of 25 women were lost to follow-up and were therefore excluded from the analysis. Among the remaining cases, medical treatment was successful in 14(73.68), with no significant correlation found between treatment success and EP location (p=1.0). Specifically, six out of nine cervical EP cases and six out of eight interstitial EP cases were successfully treated with medical management. Additionally, one case each of cesarean scar and heterotopic EP underwent medical treatment, both with successful outcomes. No significant trend was observed in the success rate of medical treatment for NTEP during the evaluated period (Cochran-Armitage test: z=0.46; p=0.644) (Figure 1).

Test Cochran-Armitage: z = 0.46; p = 0.644

Regarding the factors possibly associated with the success of medical treatment, a significant difference was only identified in relation to body weight (higher value in those with success), as shown in table 4 (p = 0.042).

**Table 4:** Comparing numerical variables and clinical treatment success

Success	Variable	n	Median	SD*	p-value**
No	Age	5	29.20	10.57	0.578
	Body weight	5	62.20	9.36	0.042
	BMI	5	23.58	3.16	0.073
	Body surface	5	1.66	0.14	0.206
	Previous number of pregnancies	5	1.00	0.71	0.088
	Gestational age (weeks)	5	7.03	2.34	0.650
	Median diameter of gestational sac	4	25.75	14.27	0.424
	HCG value at admission	4	5829.0	6236.3	0.915
	Total dose of methotrexate	5	83.30	7.12	0.226
Yes	Age	14	31.00	6.10	
	Body weight	13	79.19	24.07	
	BMI	12	29.97	9.14	
	Body surface	12	1.83	0.24	
	Previous number of pregnancies	14	2.00	1.30	
	Gestational age (weeks)	11	8.01	1.66	
	Median diameter of gestational sac	14	36.29	21.85	
	HCG value at admission	14	13812	22579	
	Total dose of methotrexate	14	123.43		

* SD – Standard Deviation

** p-value - Mann-Whitney test used for numeric values comparison between the 2 groups

Severe complications occurred in 12 cases (20) of NTEP. Among these, 5(8.3) required blood transfusion, 3(5) needed ICU admission, 4(6.7) underwent hysterectomy, and 2(3.3) required surgical reassessment. No deaths were reported. More specifically regarding the location of the EP, severe complications were observed in one case (33.3) of abdominal ectopic pregnancy, four cases (26.7) of cervical ectopic pregnancy, four cases (33.3) of cesarean scar ectopic pregnancy, two cases (8.3) of interstitial ectopic pregnancy, and one case (33.3) of heterotopic pregnancy. No severe complications occurred in ovarian EPs. No significant correlation was found between EP location and the occurrence of severe complications (p=0.272).

The occurrence of severe complications was analyzed in relation to the indications for surgical treatment. Among patients without severe complications, the most common indication for surgery was ectopic pregnancy rupture (52), followed by relative contraindication to methotrexate (36). Hemodynamic instability at admission and medical preference accounted for 4% of cases each, while other reasons contributed to the remaining 4%. In contrast, among patients who experienced severe complications, the most frequent surgical indication was hemodynamic instability at admission (33.33), followed by relative contraindication to methotrexate (22.22) and other reasons (22.22). Ectopic pregnancy rupture was the indication in 11.11% of cases, and medical preference accounted for 11.11%. A statistically significant association was found between surgical treatment indication and the occurrence of severe complications (Fisher's exact test: p = 0.015), suggesting that certain surgical indications, particularly hemodynamic instability at admission, may be linked to an increased risk of severe complications. Details are shown in table 5.

**Table 5:** Severe complication occurrence compared with surgical treatment indication motive

Variables	No n(%)	Yes n(%)
Relative contraindication to methotrexate	9(36)	2(22.22)
Rupture ectopic	13(52)	1(11.11)
Hemodynamic instability	1(4)	3(33.33)
Medical option	1(4)	1(11.11)
Others	1(4)	2(22.22)

Fisher's exact test: p= 0.015

## Discussion

EP is a common condition with a significant impact on women's health. However, limited data are available in the literature regarding NTEP and the applicability of medical treatment in this population. In this study, among the women who underwent medical treatment (n = 25), six were lost to follow-up and were excluded from the analysis, as it was not possible to confirm whether they required surgical intervention at another hospital, despite this being an unlikely scenario. The success rate of medical treatment in our study was 14(73.68), with no observed correlation between treatment success and the location of the NTEP. This success rate is comparable to the rates reported for tubal EP, which range from 70% to 95%.^(1)^ Our findings align with previous studies conducted at the same hospital, which also demonstrated high success rates of medical treatment for tubal pregnancies.^(5)^

Regarding heterotopic pregnancy, the primary treatment approach is conservative surgery, as MTX, the standard agent for medical management, can affect both the ectopic and intrauterine pregnancies.^(6)^ However, in one case in this study, medical treatment was chosen for a heterotopic pregnancy using a single dose of MTX. This approach was selected because the intrauterine pregnancy was anembryonic. Another noteworthy finding was the proportion of cervical EP cases managed surgically (3 out of 15), which contrasts with global literature trends favoring medical management.^(7)^ A detailed case analysis revealed that surgical treatment was indicated in two cases due to hemodynamic instability, while in the third case, there was a relative contraindication to MTX.

According to the medical literature, the primary factors associated with successful medical treatment include serum HCG levels at diagnosis, maximum EP diameter on ultrasound, and the presence of embryonic cardiac activity on ultrasound.^(8)^ Although data on NTEP remain scarce, existing studies suggest that the same prognostic factors influencing treatment success in tubal EP also apply to NTEP.^(9)^ In this study, the only factor significantly associated with a higher success rate of medical treatment was body weight, with greater success observed in women with higher body weight. This finding contradicts previous literature, which reports similar success rates regardless of body weight.^(10)^ It is possible that the small sample size influenced this result.

Among surgical treatment options, hysterectomy is associated with higher risks and the irreversible loss of fertility. Therefore, whenever technically feasible, conservative surgical approaches are preferred to preserve reproductive potential. Regarding medical treatment options—including single-dose MTX, multiple-dose MTX, or direct injection, the choice often depends on the experience and preference of the attending physician, as well as institutional protocol guidelines.^(11)^ At the UNICAMP Women's Hospital, the management of unusual ectopic pregnancies is individualized, considering the pregnancy location, serum HCG levels, and the patient's reproductive desires. For interstitial pregnancies, the preferred initial approach is conservative, using systemic MTX in a multiple-dose protocol, with weekly monitoring of HCG levels until negative. If clinical treatment fails, cornual resection or hysterectomy may be necessary. For cervical ectopic pregnancies, medical management with systemic MTX in a multiple-dose protocol is preferred, with weekly HCG monitoring until negative. If indicated, cervical curettage may be performed; however, due to the high risk of severe bleeding, balloon tamponade or cerclage placement should be considered, with the knot tightened only in cases of active bleeding. Hysterectomy may be necessary in cases of treatment failure or when the patient has completed childbearing.

For ectopic pregnancies in cesarean scars, conservative surgery is the first-line approach, favoring hysteroscopic or laparoscopic wedge resection to preserve fertility. This may explain why 10 out of 12 cesarean scar ectopic pregnancies in this study were managed surgically. The optimal management of cesarean scar pregnancy remains unclear, with significant variations in clinical practice worldwide. However, studies, including a systematic review of 3,127 patients, suggest that surgical treatment—comprising hysterectomy, surgical resection, and curettage—achieves higher pregnancy resolution rates than medical management (83% vs. 60%). Among surgical approaches, curettage demonstrated the lowest success rate. Additionally, a key advantage of surgical resection over other treatments is the ability to excise the scar and restore uterine integrity, potentially improving future reproductive outcomes.^(12)^ Hysterectomy may be considered if conservative treatment—either medical or surgical—fails, or if the patient has completed childbearing. For ovarian ectopic pregnancies, MTX treatment may be considered if the pregnancy is intact and HCG levels are below 5,000 IU, with weekly monitoring until negative. If surgery is required, oophoroplasty should be prioritized whenever possible to preserve ovarian function. For abdominal ectopic pregnancies, magnetic resonance imaging is recommended to accurately determine implantation site. Treatment is surgical, with pregnancy resection. Finally, for heterotopic pregnancies, systemic MTX is contraindicated due to the risk of compromising the viable intrauterine pregnancy. Management should prioritize the least invasive surgical approach possible for ectopic pregnancy removal.

Regarding the occurrence of serious complications in women with surgical indication, they were more frequent in women in whom the indication was "hemodynamic instability" or "other" than in women in whom the procedure was indicated due to "rupture ectopic" or "relative contraindication to methotrexate." This finding suggests the importance of the patient's general clinical status upon admission, more than the rupture of the gestational sac itself. In 1994, Thaddeus and Maine^(13)^ proposed the "three delays model" as a tool to identify indirect factors that contribute to maternal mortality from the onset of obstetric complications to delivery. This model identifies three critical phases that can directly impact morbidity and mortality: the delay in deciding to seek care (first delay), the delay in identification and arrival at the health facility (second delay) and the delay in receiving adequate care and treatment at the hospital. unit (third delay). The "first delay" is associated with family and community factors, such as the woman's socioeconomic status, her knowledge about the danger signs in pregnancy, her perception of the severity of the disease during pregnancy, the physical distance to the health unit, the potential costs of care and previous experience with the health system.^(14)^ The "second delay" refers to accessibility challenges, including distance, availability and cost of transport, as well as the distribution of health facilities in the woman's area of residence.^(14)^ The "third delay" is related to the services offered at the healthcare facility, which may be inadequate due to lack of supplies, lack of adequate and well-trained staff.^(15)^ Although these data are not available, it is possible to infer that one of the causes with a significant contribution to hemodynamic instability upon admission is the delay in pre-hospital care: both due to delay in seeking care after the onset of symptoms, and due to difficulty in accessing to the health service.

Although this study covers an extended period at a university hospital with significant experience in the clinical management of EP, it has certain limitations. These include the loss of follow-up in a few cases, its retrospective observational design, and the small number of NTEP cases, which reflects the low incidence of the condition. Consequently, multiple regression models could not be applied. Despite the small sample size, the findings of this study contribute to the existing body of knowledge by providing valuable insights into NTEPs. The results reinforce the need for further research in this area and highlight the importance of future studies to refine diagnostic and treatment strategies for this complex condition.

## Conclusion

This study provides valuable insights into the management of NTEP, highlighting the success rates of medical treatment and the occurrence of severe complications. The findings suggest that medical management with methotrexate is a viable option for selected cases of NTEP, with success rates comparable to those reported for tubal ectopic pregnancies. However, the presence of hemodynamic instability at admission was significantly associated with severe complications, reinforcing the importance of early diagnosis and timely intervention. Given the rarity of NTEP, larger multicenter studies are needed to further evaluate the best treatment strategies and improve clinical outcomes. Future research should also address potential barriers to timely care, aiming to reduce maternal morbidity associated with this condition.

## Data availability

: The authors did not make the data from this article available in repositories prior to submission.
